# Differential inflammatory response and cognitive deficits following sepsis in aged versus young mice

**DOI:** 10.1002/alz70855_105715

**Published:** 2025-12-24

**Authors:** Han Noo Ri Lee, Fiona E. Harrison, Julie A. Bastarache

**Affiliations:** ^1^ Vanderbilt University, Nashville, TN, USA; ^2^ Vanderbilt University Medical Center, Nashville, TN, USA

## Abstract

**Background:**

Sepsis is a dysregulated host inflammatory response to infection. Up to 70% of sepsis patients develop delirium, and the severity and duration of delirium are strong predictors for the development of Alzheimer's disease and related dementias (ADRD). Adults over 65 are at particularly high risk for sepsis‐associated delirium, but the underlying mechanisms are not well understood. This study tests the hypothesis that aged brain exhibits heightened neuroinflammation during sepsis, which correlates with persistent cognitive deficits.

**Method:**

Male and female wild‐type mice (young: 3 months; aged: 18 months) were used to investigate the effects of polymicrobial sepsis on neuroinflammation and behavioral deficits. Sepsis was induced via intraperitoneal injection of cecal slurry (CS), and sickness was monitored by assessing illness severity. CS dose was adjusted by age (young: 2.0 mg/g; aged: 1.0 mg/g) to evaluate cognitive deficits following similar severity of illness. Behavioral assays were conducted between days 3‐7 post‐infection. On day 8, plasma and brain tissue were collected and probed for pro‐inflammatory cytokines and markers of neuronal damage using ELISAs.

**Result:**

Aged mice exhibited greater susceptibility to sepsis, as demonstrated by higher mortality rates and physiological indicators of illness severity. Despite apparent recovery from illness, aged mice showed a persistent and progressive deficit in nest‐building behavior up to a week post illness, whereas young mice displayed no such impairments. Young mice exhibited heightened systemic inflammation compared to aged mice 8 days post‐CS. This differential inflammatory response was not observed in the cortex; however, an increase in KC/GRO levels was detected in the hippocampus of young mice. Notably, no significant changes in neuronal damage markers were observed in either young or aged mice following sepsis.

**Conclusion:**

This study highlights age‐related differences in sepsis outcomes, particularly in mortality, inflammation, and behavior. Contrary to the hypothesis, aged mice did not show increased inflammation either systemically or in the brain but did show persistent and progressive cognitive deficit. Instead, young mice had exacerbated inflammation without any change in functional behaviors. Together, these findings suggest the possibility that differential inflammatory response to sepsis, rather than increases per se, may underly persistent cognitive deficit following sepsis in aged brains.

